# Development of Inorganic Particle-Filled Polypropylene/High Density Polyethylene Membranes via Multilayer Co-Extrusion and Stretching

**DOI:** 10.3390/polym13020306

**Published:** 2021-01-19

**Authors:** Pilar Castejón, Marcelo Antunes, David Arencón

**Affiliations:** Poly2 Group, Department of Materials Science and Engineering, Universitat Politècnica de Catalunya (UPC BarcelonaTech), ESEIAAT, C/Colom 11, E-08222 Terrassa, Spain; pilar.castejon@upc.edu (P.C.); marcelo.antunes@upc.edu (M.A.)

**Keywords:** particle-filled multilayer membranes, co-extrusion, annealing, uniaxial stretching

## Abstract

This work is made to ascertain the effects of mineral fillers, namely calcium carbonate and talc, on the morphology and properties of multilayer polypropylene (PP)/high-density polyethylene (HDPE) porous membranes. Multilayer membranes were prepared using the three-stage Melt-Extrusion, Annealing and Uniaxial Stretching (MEAUS) process. The orientation of PP’s crystalline phase was affected by both the flow-induced crystallization and the heterogeneous nucleation promoted by the fillers. A synergistic effect was observed in the filled samples due to the generation of pores after the stretching-induced lamellae separation and the debonding of mineral fillers from the polymeric matrix. The fillers increased the porous surface, leading to an increase of permeance to air, being this effect more marked at higher filler contents. Talc showed a higher efficiency to create porous surfaces when compared to calcium carbonate. The thermal stability of the membranes increased with filler addition, as well as their stiffness and strength.

## 1. Introduction

Polymeric porous membranes have attracted much interest as separators in different applications due to their wide range of chemical and physical characteristics, low raw material cost and great number of available manufacturing processes. Recently, dry stretch methods have been developed to reduce solvent consumption and waste generation. Void nucleation and growth can occur by four different mechanisms in the stretched samples: interface separation between immiscible polymer blends, stress-induced polymorphic transitions in crystalline polymers, debonding of particle/matrix interfaces in mineral particle-filled polymers and separation of crystalline lamellar structure after uniaxial stretching [1,2,3,4,5,6,7].

The three-stage Melt-Extrusion, Annealing and Uniaxial Stretching (MEAUS) process for producing porous membranes is a solvent-free process based on the stretching of semicrystalline polymer films. This method involves three consecutive stages: (a) film extrusion, where the polymer is melt-extruded under appropriate conditions to achieve a stacked lamellar morphology, commonly known as row-nucleated structure, resulting from stress-induced crystallization; (b) thermal annealing to achieve a near-perfect crystalline phase and lamellar thickening; and (c) uniaxial stretching, where the films are highly deformed along the machine direction, first at room temperature to generate pores and then at a high temperature to enlarge pore size by increasing lamellar separation. Following this last stretching step, a heat-setting stage is applied to maintain the dimensional stability of the resulting membranes, avoiding shrinkage and keeping the mean pore size constant. The significance of the morphological characteristics and the state of crystal orientation of the extruded precursor films on the final pore structure of the membranes have been highlighted in many studies [6,7,8,9,10,11,12,13,14].

Polyolefin microporous membranes are commonly used in lithium-ion batteries as commercial separators placed between the anode and the cathode. Usually, commercial battery separators are made from polyolefins such as polyethylene (PE) or polypropylene (PP). These materials have been commonly employed to produce microporous membranes using a wet phase inversion process or a dry-stretch process, including the MEAUS process. To increase the battery cycle performance and safety, PE and PP have been combined to form trilayer PP/PE/PP separators. This kind of separator possesses a shutdown mechanism, where the intermediate PE layer with a low melting temperature closes the pores and stops ion flow through the cell when a lithium-ion battery is overcharged, and the external PP layers, having a higher melting temperature, provide the required dimensional and mechanical stability [15,16]. Finally, some characteristics of these types of separators, such as dimensional, thermal and mechanical stability, wettability to the electrolyte, as well as pore morphology and permeability, have been shown to be enhanced with the addition of a certain amount of fillers or with polymer or ceramic coatings [17,18].

Some authors have investigated the formation of multilayer microporous membranes of PP and PE using a multilayer co-extrusion process [15,16]. According to Tabatabaei et al. [15], due to the interface and the lower crystalline orientation achieved during the cast-film extrusion stage, trilayer microporous membranes of PP and high-density polyethylene (HDPE) showed a lower permeability to water vapor than the respective monolayer membranes. The authors observed a good adhesion between PP and HDPE in the porous multilayer membrane, which they related to a transcrystallization zone formed around the interface.

Organic and inorganic fillers are usually added to thermoplastics to reduce cost and enhance material characteristics. A common method for forming porous membranes considers the delamination of inorganic fillers from the polymer matrix. Some authors have investigated the formation of microporous membranes with filled PP using a stretching method, including our previous study [19]. Nakamura [4] and Nago [5] prepared microporous PP films containing calcium carbonate (CaCO_3_) particles by extrusion and biaxial stretching, and established a relationship between the size of the particles and the porous structure. Research shows that, with decreasing filler size, low tortuosity factor, high porosity and small pore size are obtained. It is known that mineral fillers can change the hydrophilic surface characteristics, enhance the mechanical and thermal stability of membranes and have a direct influence on the generated porous structure, as most fillers have some crystal nucleation ability during semicrystalline polymer cooling, especially in the case of PP [20,21,22,23,24,25]. However, experimental results have shown that it is sometimes difficult to achieve said nucleation because of the weak interfacial interactions between particles and the polymer matrix. Inorganic surface modification using coupling agents has been shown to prevent agglomeration and promote particle dispersion during processing [26].

With all these considerations in mind, this study focuses on the production of thin multilayer membranes composed of HDPE as mid layer and blends of PP and inorganic fillers, namely calcium carbonate and talc, as outer layers. We hypothesized that these fillers, commonly employed in the industry to enhance some characteristics of polymers, would lead to changes in the crystalline structure, thermal stability and mechanical and permeance properties of the porous membranes. The influence of annealing and uniaxial stretching is also investigated.

## 2. Materials and Methods

### 2.1. Materials and Composite Compounding

Commercial grades of PP homopolymer, Isplen PP020 G3E, from Repsol (Madrid, Spain), and HDPE, HDPE KT 1000 UE, from Dow (Midland, MI, USA), were used. Both materials display a characteristic linear-like molecular architecture. Isplen PP020 G3E has a melt flow index of 0.9 dg/min (measured at 230 °C and 2.16 kg according to ISO 1133 [27]), a Vicat softening temperature of 151 °C (ISO 306, method A [28]) and a yield strength of 34 MPa (ISO 527-2 [29]). HDPE KT 1000 UE has a melt flow index of 8.0 dg/min (measured at 190 °C and 2.16 kg according to ISO 1133), a Vicat softening temperature of 131 °C (ISO 306, method A) and a yield strength of 29 MPa (ISO 527-2).

Two different commercial mineral fillers were added to the outer layers of PP. By one hand, a ultramicronized calcium carbonate, Microcarb 95T, from Reverté Minerals (Barcelona, Spain), surface-treated with MgCO_3_, Fe_2_O_3_ and amino groups. This calcium carbonate has an average particle size (D_98_) of 4 µm; by the other hand, an untreated talc, Mistrocell M90, from Imerys Talc (Paris, France), with a micro-lamellar morphology and an average particle size (D_98_) of 3.3 µm. Both fillers have a Brunauer–Emmett–Teller (BET) surface area of 13.0 m^2^·g^−1^.

PP composites were compounded with different weight percentages of filler, namely 0, 5 and 10 wt%. These compounds were obtained using a co-rotating twin-screw extruder Collin Kneter 25 × 36D (Dr. Collin, Maitenbeth, Germany), with screw length/diameter relation of 36. The extrusion temperature profile from the hopper to the die ranged from 140 to 230 °C. The melt was extruded through a circular die with a diameter of 3 mm, cooled in a water bath (water kept at room temperature) and pelletized.

### 2.2. Production of Trilayer Membranes

#### 2.2.1. Co-Extrusion of Trilayer Precursor Films

Trilayer precursor films (PP/HDPE/PP layer configuration) were extruded using a lab-scale three-layer co-extrusion line (Teach-Line Collin, E20T and E16T, Dr. Collin, Maitenbeth, Germany) equipped with a rectangular die having a width of 100 mm and a gap opening of 1.9 mm. The trilayer configuration was PP (with or without filler) as outer layers and HDPE as the mid layer. The co-extrusion adapter and co-extrusion die were set at 230 °C during extrusion. At the exit of the die, slit-open air knives were used to supply air (5 bar) to both sides of the film to achieve fast cooling. Simultaneously, the film underwent uniaxial stretching along the machine direction orientation (MDO) using calendaring rolls kept at controlled temperature (room temperature). The draw ratio was kept at 70 in order to obtain a precursor film having a nominal thickness of 30 μm. The following codes will be used in the manuscript: “PP” or “neat PP” to refer to outer layers of the trilayer membranes without filler; “PP-5C” and “PP-10C” will account for outer layers with 5 and 10 wt% of CaCO_3_; and “PP-5T” and “PP-10T” will denote outer layers with 5 and 10 wt% of talc.

#### 2.2.2. Annealing and Uniaxial Stretching

Rectangular samples were directly cut from the extruded precursor films with a length of 100 mm and width of 60 mm, to be annealed (without any external stress) at two different temperatures, 120 and 130 °C, using an air-circulating oven, for 15 min. The annealed precursor films were then tightened in two grips in a tensile configuration for uniaxial stretching along the MDO using a universal testing machine SUN 2500 (Galdabini, Cardano al Campo, Italy) equipped with a climatic chamber. They were firstly stretched at room temperature (cold stage) and secondly at 125 °C (hot stage). A stretching speed of 50 mm·min^−1^ and 35% of stretching were applied during the cold stretching step; during the second step, multilayer films were stretched at 10 mm·min^−1^ up to 230% of stretching. Once the test was finished and before relieving the films from the imposed stress, all of them were kept at the same hot stretching temperature for 90 s to stabilize the porous structure. The obtained membranes had a nominal thickness of 16–18 µm.

### 2.3. Characterization

#### 2.3.1. Orientation and Microstructural Changes of the Crystalline Phase

For the assessment of the orientation of the crystalline phase of non-annealed and annealed precursor films, a Fourier-transform infrared (FTIR) spectrometer Perkin Elmer 1000 (PerkinElmer, Waltham, MA, USA), with a spectral resolution of 1 cm^−1^ under polarized beam, was used. A minimum of three samples were tested. The IR spectra were recorded within the range 600–4000 cm^−1^. Herman’s orientation function (F) is expressed as:


F = (D − 1)/(D + 2)
(1)

where the dichroic ratio, D, is the ratio of two absorption values in the two orthogonal directions, parallel (A_0_) and perpendicular (A_90_) to the reference axis MDO:


D = A_0_/A_90_ (2)

For PP, the crystalline orientation function (F_c_) of non-annealed and annealed precursor films was determined using the absorption at the wavelength of 998 cm^−1^, attributed to the c-axis crystalline phase. For HDPE, the c-axis orientation function (F_c_) was determined using the orthogonal equation:


F_a_ + F_b_ + F_c_ = 0 (3)

where F_a_ and F_b_ are the a-axis and b-axis of the unit crystal cell at the absorption wavelength of 730 cm^−1^ and 720 cm^−1^, respectively [15].

Differential scanning calorimetry was employed to analyze changes in the endothermic signals due to microstructural changes of both PP and HDPE layers from the annealed precursor films to the membranes. A DSC Q2000 calorimeter (TA instruments, New Castle, DE, USA) was employed. Samples with a mass of 6–8 mg were heated from 30 to 200 °C at 10 °C·min^−1^. To assess about the nucleating efficiency of fillers on the crystallization of PP, the filled samples were heated to 200 °C for 5 min to erase any thermal history and subsequently cooled down to 30 °C at 10 °C·min^−1^.

#### 2.3.2. Pore Morphology

A scanning electron microscope JSM-5610 (JEOL, Akishima, Japan) operating at 2 kV was employed to examine the trilayer membranes surfaces and cross-sections. The samples were first coated with a thin layer of gold in an argon atmosphere using a SCD005 Sputter Coater (BalTec, Pfäffikon, Switzerland). An image analysis program, OmniMet (Buehler, Lake Bluff, IL, USA), was used to obtain values of the pore density and porous area assuming a circular-like porous geometry.

An etching method was employed to observe the crystal arrangement of the membrane cross-section. The samples were dissolved in a 0.7 wt% solution of potassium permanganate in concentrated sulfuric acid. The potassium permanganate was slowly added to the sulfuric acid and the mixture was stirred efficiently with a magnetic stirrer until all the permanganate was dissolved. At the end of the reaction time, samples were washed following the process described by Olley and Basset [30].

The Brunauer–Emmett–Teller (BET) analysis was used for evaluating the surface area of the trilayer membranes, using a Tristar 3000 automated gas adsorption analyzer (Micromeritics Instrument Corporation, Norcross, GA, USA). It comprises two major stages: first, it is necessary to transform a physisorption isotherm into the BET plot and from it derive a value of the monolayer capacity (*n*_m_). The BET equation is conventionally expressed in the following linear form:

(4) $$\frac{\frac{p}{p_{o}}}{n(1-\frac{p}{p_{o}})}\;=\;\frac{1}{n_{m}C}+\frac{C-1}{n_{m}C}\left(\frac{p}{p_{o}}\right)$$

where *n* is the specific amount adsorbed at the relative pressure *p*/*p*_o_, *n*_m_ is the mono-layer capacity and C is a constant. Then, in the second stage, the calculation of the BET area from *n*_m_ is made by adopting an appropriate value of the molecular cross-sectional area, *σ_m_*, occupied by the adsorbate molecule in the monolayer [31].

(5) $$a_{s}=n_{m}\;N_{A}\;\sigma_{m}/m$$

where *a*_s_ is the BET surface area, N_A_ is Avogadro’s constant and *m* is the weight of the adsorbate. The method for mesopore size analysis of the trilayer membrane was proposed by Barrett, Joyner and Halenda (BJH) and can be found in [32].

#### 2.3.3. Permeance to Air

The permeance to air of the trilayer membranes was measured using a Gurley densimeter (Lorentzen & Wettre, Kista, Sweden). The permeance to air was calculated by dividing 135.5 into the time necessary for a settled volume (100 mL) of air to pass through the sample with a fixed area (0.79 cm^2^) under a pressure of 0.02 MPa, according to ISO 5636-5 [33]. Longer time values correspond to low air permeances, usually a consequence of a long and tortuous path for air transportation through the pores.

#### 2.3.4. Thermal Stability

Thermogravimetric analysis (TGA) of the trilayer membranes was carried out to evaluate the thermal stability of the membranes using a TGA/DSC 1 (Mettler Toledo, Columbus, OH, USA). Samples with a mass of 8.0 mg were heated from 40 to 700 °C at a heating rate of 10 °C·min^−1^. All tests were performed under a nitrogen atmosphere with a constant gas flow of 30 mL·min^−1^.

#### 2.3.5. Tensile Behavior

The mechanical properties of the annealed precursor films and membranes were obtained in tensile mode along MDO. Tensile tests were performed in a universal tensile machine SUN 2500 (Galdabini, Cardano al Campo, Italy) using a 1 kN load cell and a video extensometer OS-65D (Mintron, Taipei, Taiwan). Samples were cut with the dimensions established in ISO 527-3 [34]. Tensile strength and strain at break were obtained from tensile tests performed on five samples at room temperature at a crosshead speed of 50 mm·min^−1^, whereas for Young’s modulus the crosshead speed was set at 1 mm·min^−1^.

## 3. Results

### 3.1. Influence of Annealing on the Crystalline Orientation of PP’s Outer Layers

After extrusion, thermal annealing is normally used to promote further structural rearrangement and obtain a more thermodynamically stable crystal structure with reduced irregularities and increased lamellar orientation and thickness [7,8,12]. FTIR measurements carried out in the precursor films allowed to determine the evolution of the crystalline phase orientation as a function of annealing temperature and filler content for both PP and HDPE (see values of the crystalline orientation function, F_c_, presented in Table 1). As can be seen, annealing promoted an increase in the crystalline orientation function, with small differences being found with increasing annealing temperature. For further membrane production, an annealing temperature of 130 °C was selected, as a higher value of the crystalline orientation factor accounts for a more arranged row-lamellar crystalline structure, necessary before the final uniaxial stretching step [8].

### 3.2. Influence of Filler Addition on the Crystalline Orientation of PP’s Outer Layers

**Figure 1:**
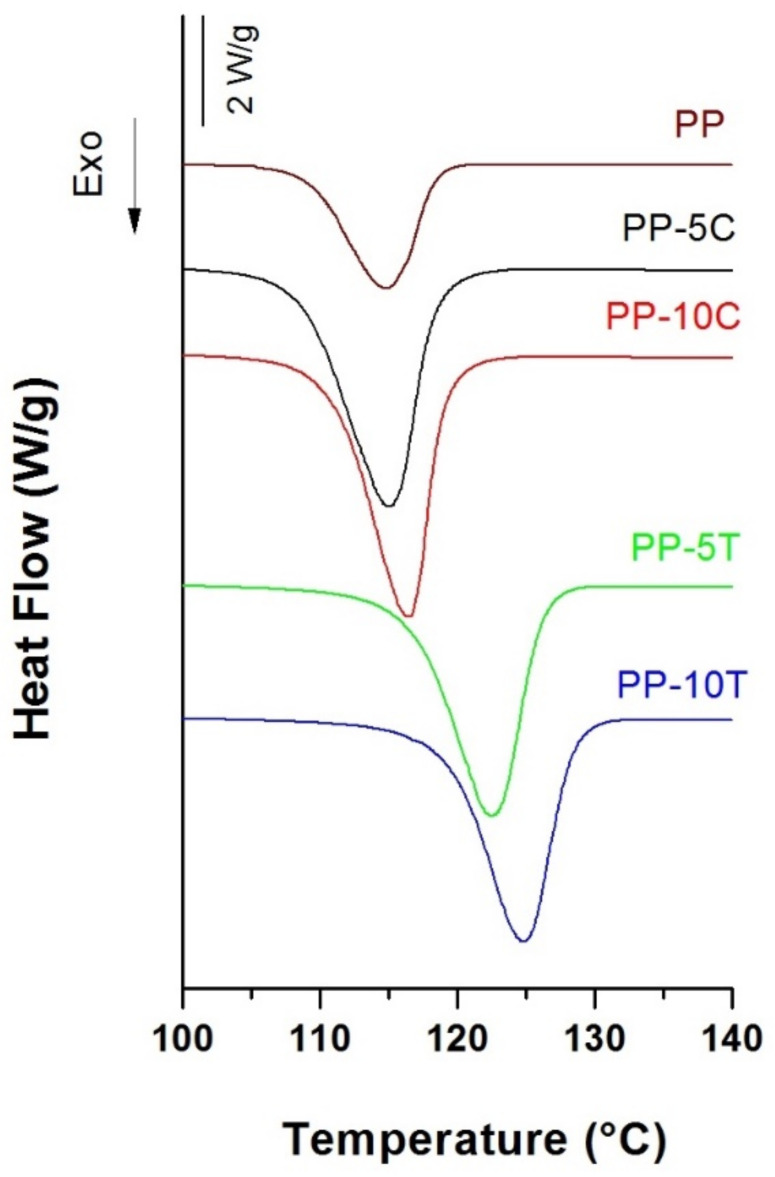
Crystallization exotherms obtained from pellets of neat polypropylene (PP), polypropylene filled with 5 wt% (PP-5C) and 10 wt% (PP-10C) CaCO_3_, polypropylene filled with 5 wt% (PP-5T) and 10 wt% (PP-10T) talc.

The outer PP-filled layers showed a higher crystalline orientation (see values in Table 1) than those reported in the monolayer films characterized in our previous work, even if annealed at a higher temperature (140 °C) [19]. It has been reported that the nucleation activity of these fillers in various composites affects the crystallization kinetics and final orientation of polymer molecules under high stresses [22,23,24]. It can be observed in Figure 1 that CaCO_3_ had a weak nucleation effect on PP’s crystallization when compared to talc [23]. The combination of the high aspect ratio and the plate-like shape of talc particles provided a higher nucleating effect and an increase in crystalline orientation.

It was also noticed that the crystalline phase orientation increased with augmenting filler content for both types of fillers. This effect was more marked when using talc. It is probable that plate-like talc particles got oriented along the extrusion flow and that the crystalline PP domain grew highly arranged perpendicular to talc’s surface [24].

### 3.3. Microstructural Changes during the MEAUS Process

**Figure 2:**
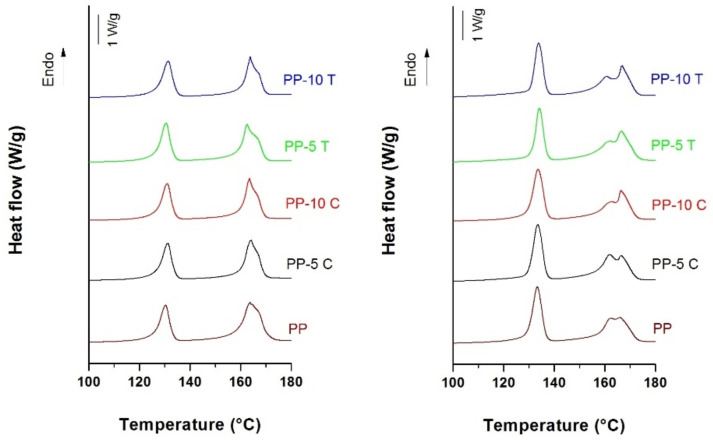
Heating thermograms of non-annealed precursor films (**left**) and membranes (**right**) with outer layers of neat polypropylene (PP), polypropylene filled with 5 wt% (PP-5C) and 10 wt% (PP-10C) CaCO_3_, polypropylene filled with 5 wt% (PP-5T) and 10 wt% (PP-10T) talc.

Endotherm signals, shown in Figure 2, provide interesting information about eventual microstructural changes during the transition from the stage of extrusion of the precursor films to the uniaxial stretching stage. HDPE melting peak resulted narrower without any additional “shoulder”, indicating the presence of a uniform crystal size structure. On the contrary, a peak and a small shoulder at high temperatures were observed for PP. The presence of this double melting peak in the non-annealed precursor films has been attributed to the presence of lamellae and fibrils (shish crystals) that have a melting temperature much higher (about 5–8 °C) than that of the lamellar crystals [11].

For the membranes, a similar but more intense right peak was also observed at high temperatures (the shoulder observed in the precursor films transforms into a clearly defined independent peak), which was related by several authors to the molecular orientation induced by uniaxial stretching. During the cold and hot stretching steps, pores are created and enlarged due to the stretching of short and long tie chains. This results in local crystallization, explaining the appearance of the so-called “interconnected bridges” that join main crystal lamellas [7,8,9,10,11,12,13,14]. Hence, it was assumed that these interconnected bridges acted like shish or fibril crystal structures located between crystal lamellae [11].

With respect to any possible effect of filler addition, it was not clearly revealed in the melting peaks; as a consequence, it was assumed that the mean lamellar thickness was similar for all membranes. Only for the talc-filled PP membranes the right peak gets more pronounced, suggesting the presence of more fibrils or interconnected bridges in the stretched films. Once again, these crystalline morphology differences are thought to come from the high molecular orientation achieved along talc’s plate-like surface.

### 3.4. Morphology and Permeance to Air of the Membranes

**Figure 3:**
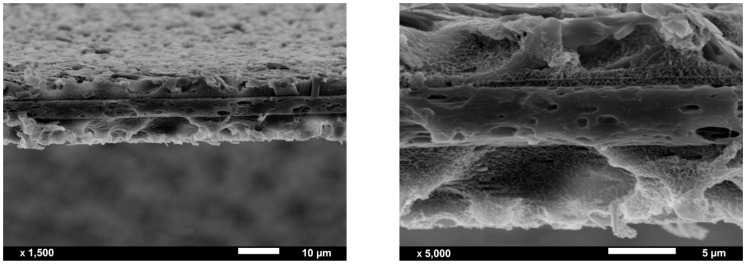
Scanning electron microscope (SEM) micrographs of the cross-section of an etched multilayer membrane. Outer layers consist of polypropylene filled with 10 wt% talc (PP-10T).

Co-extrusion may offer a significant degree of adhesion between intermediate and outer layers in multilayer films, as layers are joined in a molten state at high temperature, when using faster cooling and high drawing ratios [35,36]. In addition, in some non-compatible polymer systems, tie layers or adhesive layers can strongly bind structures together [35]. In this work, no tie layers were employed to join the PP and HDPE layers. Despite this, as can be seen in the cross-section micrographs presented in Figure 3, an acceptable adhesion was found in the multilayer membranes prepared in this study, as there was no evidence of delamination either in the scanning electron microscope (SEM) micrographs nor in the visual inspections performed during extrusion and film stretching.

It was revealed that the HDPE intermediate layer had larger pores than the outer PP layers. However, due to the melt flow properties of the selected HDPE (high melt flow index), the formation of extended chain threads or fibril nuclei in a row nucleated structure was reduced, as well as the orientation of the crystalline structure [37]. This promoted the formation of a weaker crystalline network connection between fibrils and resulted in a larger, less uniform pore structure.

**Figure 4:**
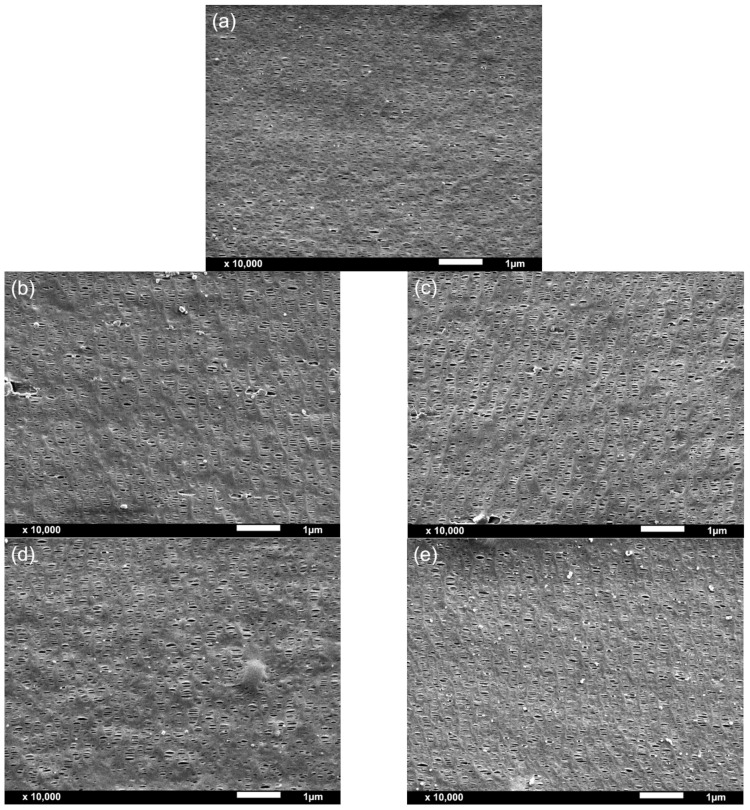
Representative SEM micrographs of the outer layer surfaces of the trilayer membranes. (**a**) Neat polypropylene (PP), (**b**) polypropylene filled with 5 wt% CaCO_3_ (PP-5C), (**c**) polypropylene filled with 10 wt% CaCO_3_ (PP-10C), (**d**) polypropylene filled with 5 wt% talc (PP-5T), (**e**) polypropylene filled with 10 wt% talc (PP-10T).

**Figure 5:**
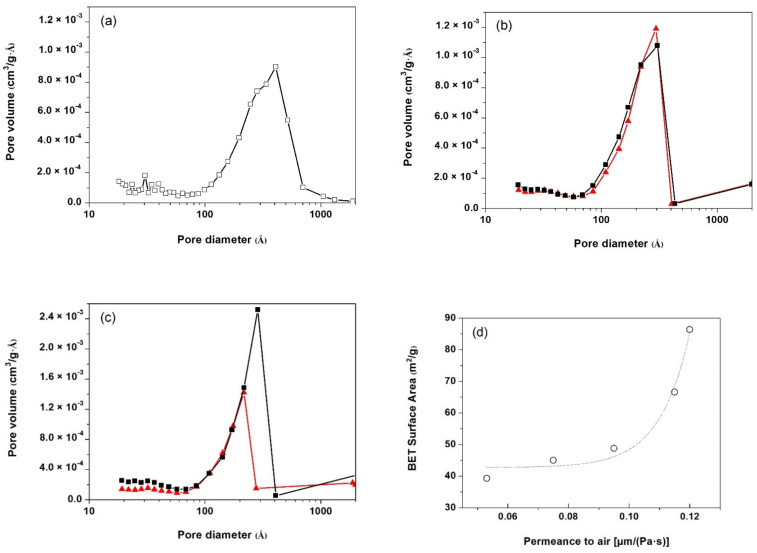
Pore volume distribution in trilayer membranes with outer layers consisting of: (**a**) neat polypropylene (PP), (**b**) polypropylene filled with calcium carbonate (PP-C) and (**c**) polypropylene filled with talc (PP-T). Filler content: <▲ 5 wt%>, <■ 10 wt%>; (**d**) correlation between the permeance to air and Brunauer-Emmett-Teller (BET) surface area of the 5 membranes analysed.

From the morphological analysis carried out by SEM (Figure 4) and BET (Figure 5), the porous density, area, pore diameter and specific surface area of the membranes were obtained (Table 2). All membranes presented a unimodal pore distribution. The addition of the fillers led to an overall increase in pore density, porous area and BET surface area, also increasing the volume of pores, ultimately resulting in an enhancement in the permeance to air. As seen in Figure 5d, the permeance to air and BET’s specific surface area showed an exponential correlation.

While calcium carbonate-filled membranes displayed both an increase in pore density and porous area by SEM analysis and in BET and BJH specific areas and volume of pores and pore diameter with increasing the amount of calcium carbonate from 5 to 10 wt%, easily explaining the higher permeance to air of PP-10C membrane when compared to PP-5C membrane, PP-10T membrane, although once again displaying a higher permeance than PP-5T, showed apparently lower values of pore density and porous area. At this point, it has to be said that the values of pore density and porous area resulting from SEM analysis are quite approximate (they were calculated assuming a circular-like porous geometry), explaining the necessity of having to perform BET and BJH analyses in order to have a more accurate explanation of the permeance values of the membranes. It is seen in Table 2 that the PP-10T membrane displays significantly higher values of BET and BJH surface areas and volume of pores and pore diameter than PP-5T, explaining the higher permeance of PP-10T membrane with augmenting talc content.

In polymer-filled membranes, pore nucleation and growth can occur by the debonding of particles from the polymer matrix upon uniaxial stretching. The synergistic effect from the combination of filler debonding from the polymer matrix under stress and pore formation by stretching-induced lamellae separation results in an increased porous surface and high membrane permeance. The possible presence of large agglomerates at high filler content is the most plausible cause that explains the increase of permeance with augmenting filler amount. Comparing the more precise BET and BJH analyses results, talc particles had a more remarkable effect than calcium carbonate particles, as this type of filler probably promoted a different crystalline structure with a high orientation degree, leading to a high porosity and pore interconnectivity.

### 3.5. Thermal Stability of the Membranes

**Figure 6:**
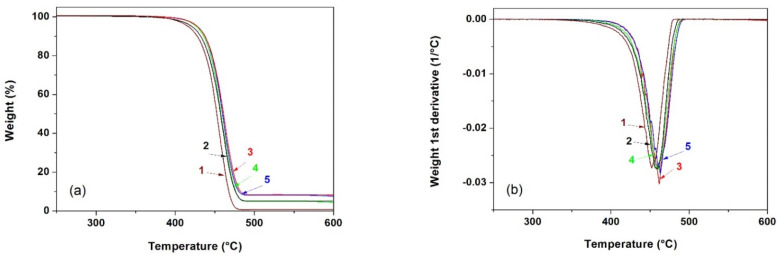
(**a**) Thermogravimetric analysis (TGA) and (**b**) Derivative thermogravimetric (DTG) curves showing the thermal decomposition of the membranes with outer layers consisting of: (1) neat PP, (2) polypropylene filled with 5 wt% CaCO_3_ (PP-5C), (3) polypropylene filled with 10 wt% CaCO_3_ (PP-10C), (4) polypropylene filled with 5 wt% talc (PP-5T), (5) polypropylene filled with 10 wt% talc (PP-10T).

Figure 6 shows the weight loss curves of the different membranes obtained by thermogravimetric analysis. From these curves, it was possible to obtain the values collected in Table 3, particularly the temperatures corresponding to a mass loss of 10% (*T*_0.1_) and 50% (*T*_0.5_), the relative mass loss at 400 and 600 °C and the temperature corresponding to the maximum decomposition rate (*T*_max_).

Several studies have demonstrated that under an inert nitrogen atmosphere, TGA curves for HDPE and PP display a single step degradation process in which the mass loss is greater between 350 and 450 °C. The thermal degradation mechanism for both HDPE and PP involves a primary degradation pathway that initiates via molecular random scission, a propagation or free radical transfer process and a termination step [38,39]. Neat multilayer membranes started to decompose at around 400 °C under an inert nitrogen atmosphere. This temperature shifted about 7–14 °C to higher values for the mineral-filled membranes. Regarding the temperatures corresponding to a 10 and a 50% mass loss and T_max_, they increased with augmenting filler content.

The observed increase in thermal stability for the multilayer membranes is thought to be due to several factors: on the one hand, the high barrier properties of the mineral particles and, secondly, the restriction of the mobility of polymer molecules due to the presence and interaction with filler particles, especially in the particles’ vicinity, the combination of which causing a delay in the volatilization of the decomposition products. It has been reported that this type of fillers has a much higher thermal conductivity than the polymer substrate [3,22], facilitating heat dissipation and, therefore, avoiding heat accumulation at a certain point, promoting the beginning of polymer degradation at a slightly higher temperature.

### 3.6. Mechanical Characterization of the Annealed Precursor Films and Membranes

**Figure 7:**
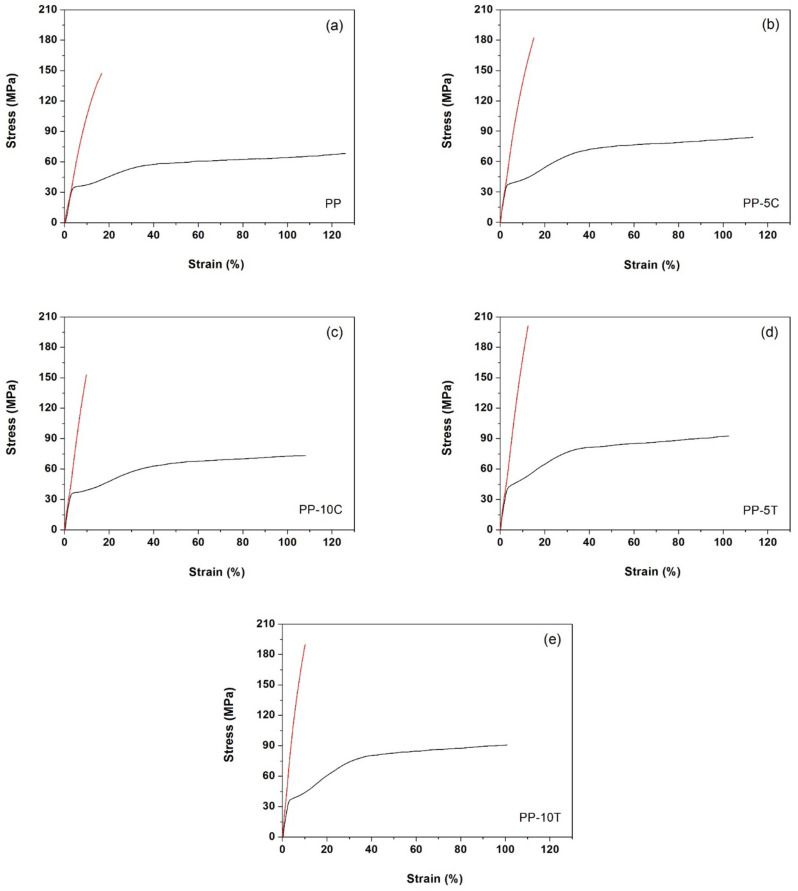
Stress-strain curves of the annealed precursor films (black line) and membranes (red line), with outer layers consisting of: (**a**) Neat polypropylene (PP), (**b**) polypropylene filled with 5 wt% CaCO_3_ (PP-5C), (**c**) polypropylene filled with 10 wt% CaCO_3_ (PP-10C), (**d**) polypropylene filled with 5 wt% talc (PP-5T), (**e**) polypropylene filled with 10 wt% talc (PP-10T).

Tensile testing has been used to determine and compare the mechanical properties of the annealed precursor films and membranes. As can be seen in Figure 7, a dramatic change on the stress–strain behavior is observed for all materials. Membranes showed a build-up in terms of stiffness and ultimate tensile strength with a significant reduction in ductility (Table 4). This behavior is explained on the basis of the high molecular orientation along MDO that is generated during the uniaxial stretching step of the MEAUS process.

Regarding the influence of filler addition, the presence of the rigid particles caused a reduction in molecular mobility, leading to increasingly lower strain at break values for filled membranes with increasing filler amount, influenced by the polymer-filler interactions and polymer chain confinement [40,41,42,43]. The ultimate tensile strength of the filled membranes decreased with augmenting filler content, which could be explained by the formation of larger particle aggregates (as found in SEM analysis).

These results revealed a competition between self-reinforcement mechanisms and damage mechanisms by debonding in the mineral-filled membranes. The presence of large particle agglomerates leads to stress concentrations in the matrix, which are detrimental to mechanical performance since they could act as stress raisers and promote premature cracking. The reinforcing mechanism of mineral fillers is strongly linked to the good interfacial adhesion between the polymer matrix and the embedded particles. In addition, a good interfacial adhesion gives a more stable and uniform filler dispersion during compounding. We hypothesize that particle surface treatments and the use of nanosized particles could improve the interaction of talc and CaCO3 with PP’s matrix, leading to filled membranes with increased tensile strength at higher filler contents.

## 4. Conclusions

In this study, the structure and performance of trilayer porous membranes made from HDPE as mid layer and PP filled with CaCO_3_ or talc as outer layers have been investigated. Crystallization and crystalline orientation were affected by both the flow-induced crystallization and the nucleating effect of fillers. Compared to CaCO_3_, plate-like talc particles seemed to have been oriented along the melt-flow direction. The addition of talc under extensional flow and resulting orientation promoted the transcrystallization of PP on the filler’s surface, increasing the crystalline orientation of PP.

The analysis of the porous structure revealed that a higher filler content promoted extensive debonding of the fillers from the polymer matrix, plus pore formation by stretching-induced lamellae separation resulted in membranes with a higher volume of bigger pores and hence with a higher permeance to air. Talc and calcium carbonate enhanced the thermal stability of the membranes. Compared to the annealed precursor films, membranes showed a pronounced increase in stiffness, tensile strength and a drastic decrease in ductility along MDO. Filler addition showed a similar trend, with tensile strength reaching a maximum value at 5 wt% of filler.

## Figures and Tables

**Table 1:** Crystalline orientation function (F_c_) for polypropylene (PP) and high-density polyethylene (HDPE) layers in annealed and non-annealed (NA) precursor films. PP-C and PP-T stands for polypropylene outer layers containing calcium carbonate and talc respectively. Standard deviation was lower than 5%.

Annealed Precursor Films	Filler Content (wt%)	Annealing Temperature (°C)	F_c_
HDPE mid layer	0	NA	0.58
		120	0.60
		130	0.63
Unfilled PP outer layers	0	NA	0.46
		120	0.58
		130	0.60
PP-C outer layers	5	NA	0.45
		120	0.59
		130	0.66
	10	NA	0.48
		120	0.63
		130	0.67
PP-T outer layers	5	NA	0.70
		120	0.78
		130	0.79
	10	NA	0.74
		120	0.87
		130	0.87

**Table 2:** Parameters obtained from scanning electron microscope (SEM) and Brunauer–Emmett–Teller (BET)–Barrett, Joyner and Halenda (BJH) analyses, and Gurley tests carried out on trilayer membranes. Membrane code refers to the outer PP-based layers.

Membrane	Filler Content (wt%)	SEM		BET and BJH				Permeance to Air (µm·Pa^−1^·s^−1^)
		Pore Density (Pores·μm^−2^)	Porous Area (%)	BET Surface Area (m^2^·g^−1^)	BJH Surface Area (m^2^·g^−1^)	Volume of Pores (cm^3^·g^−1^)	Pore Diameter (µm)	
PP	0	14.0	4.8	39.3	45.8	0.38	0.034	0.039
PP-C	5	18.3	5.9	45.0	52.1	0.43	0.033	0.075
	10	20.9	5.9	48.8	57.8	0.57	0.039	0.086
PP-T	5	23.9	7.8	66.6	78.6	0.58	0.029	0.115
	10	23.3	5.9	86.4	98.1	0.81	0.033	0.120

**Table 3:** Thermogravimetric analysis results. Membrane code refers to the outer PP-based layers.

Membrane	Filler Content (wt%)	*T*_0.1_ (°C)	*T*_0.5_ (°C)	Mass Loss at 400 °C (%)	Mass Loss at 600 °C (%)	*T*_max_ (°C)
PP	0	425.5	452.5	1.8	99.1	452.7
PP-C	5	436.1	457.8	1.5	95.3	457.5
	10	438.3	460.4	0.6	91.7	464.2
PP-T	5	432.1	456.8	0.8	94.7	461.2
	10	437.4	460.9	0.5	91.6	462.9

**Table 4:** Tensile properties of the annealed precursor films and membranes. Sample reference stands for outer layers. Standard deviation between brackets.

Sample	Filler Content (wt%)	Young’s Modulus (GPa)		Ultimate Tensile Strength (MPa)		Strain at Break (%)	
		Annealed Precursor Film	Membrane	Annealed Precursor Film	Membrane	Annealed Precursor Film	Membrane
PP	0	1.35 (0.07)	2.19 (0.05)	69.5 (4.9)	140.1 (6.8)	125.3 (14.2)	20.1 (1.0)
PP-C	5	1.79 (0.09)	2.63 (0.08)	84.4 (10.7)	181.9 (7.6)	112.5 (18.3)	15.9 (1.4)
	10	1.80 (0.07)	2.60 (0.06)	71.1 (8.8)	152.5 (9.9)	108.7 (20.1)	10.9 (1.9)
PP-T	5	1.80 (0.07)	2.80 (0.07)	92.9 (10.1)	198.2 (9.5)	99.3 (21.0)	14.3 (1.3)
	10	1.82 (0.08)	2.81 (0.09)	87.9 (8.3)	189.4 (9.0)	98.1 (15.2)	10.6 (1.6)

## Data Availability

The data presented in this study are available on request from the corresponding author.
